# Medical Items

**Published:** 1913-02

**Authors:** 


### MEDICAL ITEMS

### SURGICAL SUGGESTIONS.

Splinters of hard wood, like pieces of glass, may become encysted in the tissues, and can often be drawn out whole by one end. But soft wood, and especially old wood, breaks on traction, and unless the wound is made large enough to expose it all, even very large fragments may be left, unrecognized, in the tissues.

—*American Journal of Surgery.*

---

Ichthyol is helpful in the treatment of chronic non-suppurating paronychia. The underlying cause of the affection must be sought—syphilis, eczema, or favus of the nail, the use of caustic alkalies on the hand, etc.

*American Journal of Surgery.*

---

Hemolysis tests are desirable before selecting a donor for transfusion, but if the case is of great emergency this may be dispensed with, since hemolysis is unlikely if the donor is free from malignant growth and tuberculosis. Syphilitic taint must of course be excluded.

*American Journal of Surgery.*

